# Radiomodulation—The Final Frontier of Radiosurgery?

**DOI:** 10.3390/brainsci16070751

**Published:** 2026-07-15

**Authors:** Fred C. Lam, Evan Chau, Yazhen Shi, Bryan Martinez, Amar Hamdan, Jay L. Gill, Nirmeen Zagzoog, Neeraj Kalra, Yusuke S. Hori, Michael B. Schneider, John Adler, David J. Park, Steven D. Chang

**Affiliations:** 1 Department of Neurosurgery, Stanford University School of Medicine, Stanford, CA 94305, USA; fredlam@stanford.edu (F.C.L.); ammar2000ham@gmail.com (A.H.); jlgill@stanford.edu (J.L.G.); nzagzoog@stanford.edu (N.Z.); nkalra@stanford.edu (N.K.); yshori@stanford.edu (Y.S.H.); djpark@stanford.edu (D.J.P.); 2 Department of Neuroscience, University of California, Berkeley, Berkeley, CA 94720, USA; evan.chau@ucsf.edu; 3 Department of Natural Sciences, Pasadena Community College, Pasadena, CA 91106, USA; marsshi13@gmail.com (Y.S.); bryanroelmartinez@gmail.com (B.M.); 4 Department of Psychiatry, Stanford University School of Medicine, Stanford, CA 94305, USA; mbschneidermd@gmail.com

**Keywords:** stereotactic radiosurgery, CyberKnife, Gamma Knife, proton beam radiation therapy, conventional radiotherapy, neurosurgery, functional neurosurgery, neuropsychiatry, psychiatry, DNA damage response, depression, pain, obesity, obsessive–compulsive disorder, addiction

## Abstract

**Highlights:**

**What are the main findings?**

- Delivery of focused ionizing radiation to specific brain loci and nerves modulate their local microenvironment and neuronal milieu, leading to neuromodulation.
- This modulatory effect leads to downstream signaling alterations across neuronal networks.

**What are the implications of the main findings?**

- Understanding how delivery of focused radiation can achieve neuromodulatory effects in the brain opens up novel avenues for treating a multitude of neurologic disorders including tremors, chronic pain syndromes, and psychiatric illnesses.
- Rigorous translational research is needed to identify the neurobiological effects of focused radiation on neural processing centers in order to understand the clinical effects in humans.

**Abstract:**

Stereotactic radiosurgery (SRS) delivers high doses of focused ionizing radiation (IR) to a defined target while sparing surrounding tissues. The delivery of focused doses of IR has proven to be an effective modality for the treatment of brain tumors, cerebrovascular lesions, and primary neuropathic pain conditions. More recently, the emerging concept of “radiomodulation” to rewire neural circuitry through the delivery of focused IR to specific neural relay centers has emerged as an alternative way to treat neurological conditions, such as essential tremor, trigeminal neuralgia, and psychiatric illnesses. In this article, we performed a scoping review of the existing data supporting the ability of focused doses of ionizing radiation to achieve modulation of neural circuits for the treatment of neurological conditions. We review the current understanding of the neurophysiological mechanisms of radiomodulation, the gaps in knowledge limiting its widespread use for in-human applications and stress the unmet need for ongoing research to rigorously prove that radiomodulation may be the “final frontier” as a non-invasive, non-pharmacological, versatile, and tunable modality for the treatment of a multitude of neurological conditions.

## 1. Introduction to the History of Radiomodulation

Dr. Lars Leksell, largely regarded as the inventor of stereotactic radiosurgery (SRS), initially intended SRS to be used for the treatment of movement disorders such as Parkinson’s Disease, facial pain due to trigeminal neuralgia (TN), and neuropsychiatric illnesses [1]. Evidence that delivery of focused IR could affect neural circuits was demonstrated in some of the first patients whom Dr. Leksell treated using SRS in the early 1950s who were suffering from TN, by targeting the trigeminal ganglion in Meckel’s cave [2]. Early use of SRS for the treatment of intractable neuropathic pain by targeting the thalamus [3] and treatment-refractory obsessive–compulsive disease (OCD) by targeting the ventral limb of the internal capsule [4] reported neurotoxicity due to higher than necessary doses of radiation.

**Figure 1:**
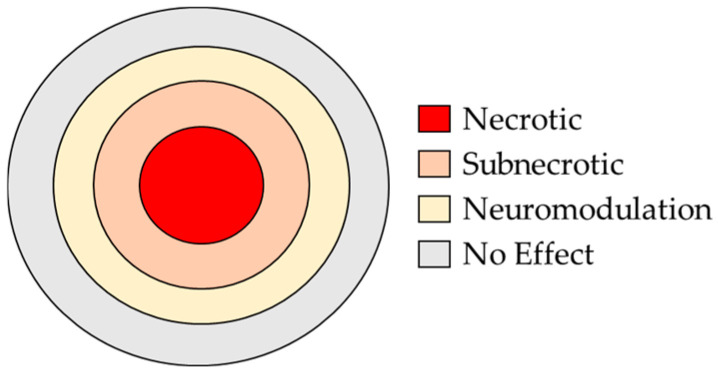
The “Cockade” theory of SRS-induced neuromodulation on normal brain. Following SRS treatment, four concentric “zones” are observed, including (1) a central histologic “necrotic” zone; (2) a “subnecrotic” zone where cell death is observed with a dying off of glial cells and preservation of non-cycling neurons; (3) a “neuromodulatory” area where the radiomodulatory effects of SRS is demonstrated; and (4) a peripheral “no effect” area of histological and functional normal brain.

As the field began mapping the normal tissue dose constraints of different regions of the brain, radiosurgeons began observing that patients with arteriovenous malformations (AVMs) located in seizure-prone eloquent cortex had an 85% chance of seizure control following SRS treatment of their AVMs even prior to complete radiographic occlusion of flow through the nidus of the AVMs [5,6,7,8,9]. Regis and colleagues were the first to report the biochemical changes in the local neuronal milieu leading to differential functional effects following Gamma Knife SRS for the treatment of mesial temporal lobe epilepsy [10,11], proposing the “cockade” theory summarizing the effect of an SRS dose on the normal brain by identifying four concentric zones. The center “necrotic” zone is surrounded by the “subnecrotic” area where differential SRS-induced cell death is observed with a dying off of glial cells and preservation of non-cycling neurons. The periphery around the “subnecrotic” zone is the “neuromodulatory area”. The area outside the “neuromodulatory area” has no observable effects (Figure 1).

Regis and colleagues further postulated that several factors contributed to neuromodulatory effects, including biochemical and cellular changes and the genetics of the patient. Diffusion-weighted imaging showed edema in subcortical white matter tracts and signs of myelin sheath splitting and periaxonal space enlargement. Treatment of mesial temporal lobe epilepsy with SRS caused major changes in the white matter tracts that extended far beyond the treatment target zone, along association fiber tracts [11,12]. However, imaging changes on MRI do not always accurately portray propagation of protein messenger signaling along fiber tracts, thus necessitating the need for ongoing basic science research to decipher the mechanisms underlying the neuromodulatory effects of SRS to in order to better under the clinical efficacy of radiomodulation [13]. The tissue sparing property of SRS makes it an ideal modality to treat central nervous system (CNS) conditions [14], with well born out mechanisms of acute and long-term toxicity of radiation-induced CNS injury [15,16], and accurate calculations of normal tissue dose constraints around organs at risk [17].

In this scoping review, we present preclinical evidence detailing the cellular and neurophysiological mechanisms thought to contribute to the neuromodulatory effects of dose-dependent effects of focused radiation. We then describe the emerging use of SRS to achieve neuromodulation for the treatment of patients with tremors, chronic pain disorders, and neuropsychiatric illnesses and report on the current limitations and controversies of using SRS to achieve neuromodulation in humans. As radiomodulation clinical trials are currently still in their infancy, we will have to wait for larger-scale, longitudinal follow-up in order to understand the clinical safety and efficacy of SRS for the treatment of an ever-growing list of non-oncological neurological conditions.

## 2. Methods

We performed a scoping review on the topic of radiomodulation by performing a PUBMED search from the dates ranging from 1 January 1930–31 May 2026 using the MeSH search terms ‘neuromodulation’, ‘radiosurgery’, ‘radiomodulation’. The starting year range was set at 1930 to adequately cover the historical timeline of the invention of radiosurgery and the first reports of neuromodulation using focused radiation. Search results returned 274 articles. Articles that solely discussed the use of radiosurgery for the treatment of brain tumors were excluded. We included articles that discussed the purported mechanisms of low-dose focused radiation on the brain in preclinical animal models, articles that discussed the use of radiosurgery for the treatment of functional neurosurgical conditions, pain disorders, and psychiatric disorders. To avoid bias, we included articles that compared the effects of low-dose focused radiation across all of the available radiosurgical devices on the market, including Gamma Knife, CyberKnife, and ZAP. We also included articles that discussed the limitations and controversies surrounding the current use of radiosurgery to treat functional and psychiatric disorders. For evidence grading, we excluded single case reports and included case series, systematic reviews, and meta-analyses. Following these inclusion and exclusion criteria, 158 articles were selected as the foundation of this scoping review.

## 3. Preclinical Animal Studies of Radiomodulation

Early studies exposing animals to low to moderate doses of radiation provided strong evidence of radiomodulation. Whole-body animal exposure to radiation ranging from 50 to 400 rads modified gustatory sensation [18] and lowered seizure thresholds in rats [19]. Exposure to doses ranging between 1000 and 1500 rads produced arousal and increased locomotion [20,21,22], while exposure to >10,000 rads caused lethargy, ataxia, disorientation, and reduced locomotion [23,24,25].

**Figure 2:**
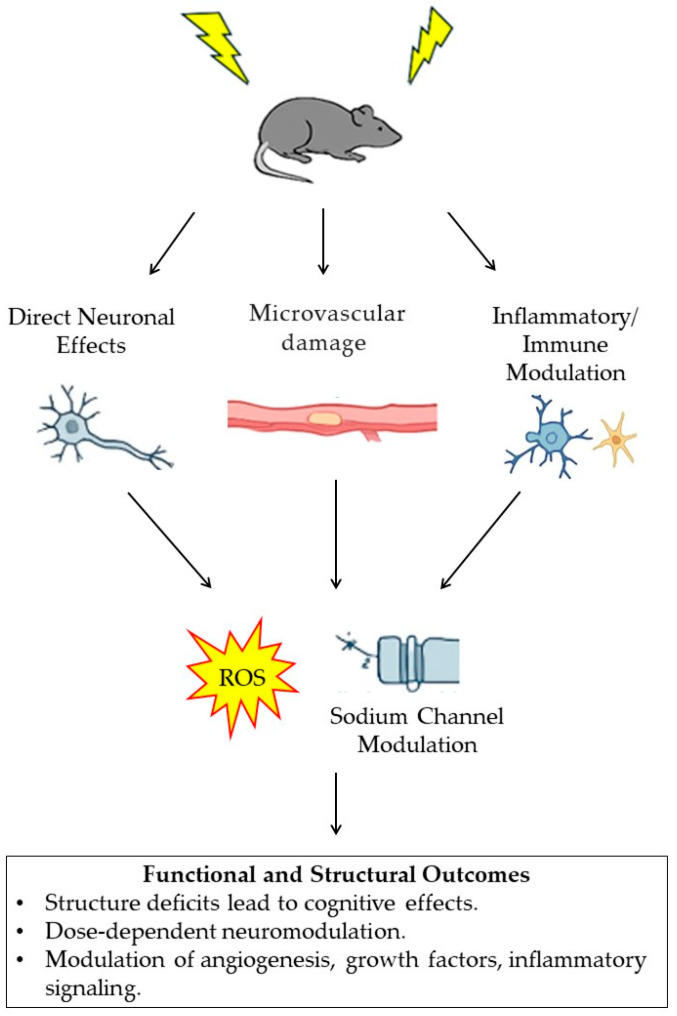
Common mechanistic pathways of radiation-induced neural and neurovascular dysfunction. Schematic overview illustrating how radiation exposure induces converging effects in the central nervous system, including direct neuronal modulation, vascular and endothelial changes, and neuroinflammation with glial activation (astrocyte IL-6–JAK–STAT3 signaling, microglial interferon-driven activation, and neurovascular unit dysregulation). Secondary molecular effects, including oxidative stress, sodium channel modulation, and stochastic segmental damage, further influence neuronal excitability and network function. Together, these processes underlie region- and dose-dependent functional and structural outcomes of radiomodulation.

Focal doses of ionizing radiation have been shown to affect neuronal activity in a manner that does not result in cell death. In vivo data have shown that exposure to 40–60 Gy of ionizing radiation resulted in reduced spontaneous neuronal firing and synaptic function in rodent models while leaving histologically intact tissue, but high doses (≥80 Gy) caused axonal degeneration and necrosis [26,27]. Mechanistically, radiomodulation may also involve neuronal hyperpolarization, blockade of voltage-sensitive sodium channels and indirect effects mediated by glial cells, alteration of blood vessels and changes in the extracellular matrix (Figure 2) [28,29,30]. Acute high-dose radiation induces oxidative stress and accumulation of reactive oxygen species (ROS) in differentiated neural cells (Figure 1) [31]. These studies point to a very promising therapeutic pathway, as radiation and radiomodulation can attenuate and down-regulate hyperactive neural circuits without causing permanent tissue injury. This outlines a basis for developing radiomodulation as a clinical treatment for conditions such as trigeminal neuralgia and epilepsy [13].

### 3.1. Synaptic Neuromodulation Following Low-Dose Radiation Exposure

A seminal paper published by Pellmar and Lepinski in 1993 studied the electrophysiological effects of exposing guinea pigs to whole-body gamma-irradiation equivalent to midline tissue doses of either 5 Gy or 10 Gy [32]. The pigs were euthanized at 30 min, 1 day, 3 days, and 5 days after irradiation or sham irradiation, their brains removed, 400- to 450-micron thick hippocampal slices were prepared, and patch clamping recordings performed to detect dose-dependent and change electrophysiological signaling between irradiated and sham irradiated animals. The results showed dose-dependent decreases in spike amplitude production which recovered at variable levels within several days, demonstrating for the first time a neuromodulatory phenotype following low-dose radiation exposure to the brain. They further demonstrated in the same study that greater than 40 Gy of X-ray exposure led to increased postsynaptic potentials at the expense of reducing spike generation, allowing for functional neuromodulation in the absence of overt neuronal cell death. These results suggest that IR has dose-dependent effects on synaptic integration and neuronal excitability [32].

In parallel, Mullin and colleagues showed that electrons or γ-rays directly inhibited voltage-activated sodium channels in isolated rat brain synaptosomes, causing dose-dependent lowering of maximal sodium ion uptake without any decrease in membrane fluidity, confirming further that this hypothesis is a mechanism of direct channel or protein modification and not lipid mediated [30]. They also reported that veratridine-stimulated ^22^Na^+^ uptake decreased in a dose-dependent manner, with the maximal effect diminished while the affinity of veratridine for its binding site remained unchanged, indicating a selective impairment of channel function. The inhibitory effect was greatest over the first few seconds of channel activation at the peak moment of sodium influx. The sensitivity to radiation was different depending on neurotoxins: batrachotoxin-stimulated uptake was less affected and [^3^H]saxitoxin binding remained unaltered, suggesting that the structural integrity of the channels was generally maintained. Furthermore, radiation did not disturb the enhancement of veratridine-stimulated uptake by synaptic vesicle polypeptides and did not change membrane fluidity, which further reinforced the assumption that IR acted directly on the channel protein rather than the surrounding lipid milieu. These results collectively corroborate that IR targets sodium channel proteins and serves as a protein- or channel-specific modulator [30].

In addition, acute radiation effects on neurons have been observed in ex vivo and in vivo electrophysiological models. In guinea pigs, when subjected to 5–65 Gy of X-rays in hippocampal slices, synaptic signaling was rapidly affected in a time- and dose-dependent manner. It is interesting to note that doses ≥40 Gy improved postsynaptic potential magnitude with a damping effect on population spike responses, indicative of faulty neuronal activity despite induced synaptic depolarization. The effects were induced during and shortly post-irradiation and there was no recovery during the period studied, which reflects a direct and persistent disruption of hippocampal network function at high doses [33]. Similar to the data derived from other invertebrates, species- and system-dependent resistance is shown by the squid giant-fiber system: focal irradiation of 140–300 Gy on the stellate ganglion resulted in almost intact synaptic transmission and action potential propagation, and only minimal change in refractory periods and spike kinetics, suggesting that large-caliber axonal systems are resilient to acute radiation [34].

In mouse and rat hippocampal models, the presence of radiation in the low dose range of 0.1–10 Gy leads to progressive reductions in dendritic branching, spine density, and neurogenesis. Changes vary based on age, strain, and type of radiation and occur in a subacute manner over a period of 10–42 days. This structural remodeling may also restrict the safe therapeutic window following radiomodulation and contribute to long-term cognitive changes [35]. Long-term studies have provided evidence for the possibility that initial modulatory action could evolve to delayed synaptic dysfunction. In mice exposed to ^56^Fe particles, suppression of long-term potentiation weakened prematurely, while synaptic output gradually declined in a dose-dependent manner over 6 to 12 months [36].

### 3.2. Neurovascular and Inflammatory Responses After Stereotactic Radiosurgery

Modern focal irradiation models using SRS are used to characterize the local vascular and inflammatory abnormalities relevant to clinical radiosurgery. Doses of 15 to 60 Gy in one hemisphere will lead to dose-dependent microvascular damage, astrocyte hypertrophy, blood–brain barrier disruption and activation of microglia cells. In a spatially resolved transcriptomics application, they discovered that neurovascular unit responses are coordinated to many signals. In astrocytes, they are involved in this inflammatory pathway using IL6–JAK–STAT3 mediation, while microglia activation increased in response to interferons. These findings provide evidence that radiomodulation is not only an act of nerve cells but also implicates a diversity of brain cells and their associations [37].

Preclinical studies also showed that stereotactic irradiation can induce a progressive cascade of vascular and parenchymal changes. Constanzo and colleagues irradiated the primary somatosensory and motor cortices of rats with a single Gamma Knife dose and monitored changes with MRI and histology [38]. No immediate vascular alterations were detected, but after 54 days, small necrotic lesions were observed with surrounding vascular permeability. Between day 54 and 110, the necrotic region grew and increased, which formed a ring-like structure with areas of mixed tissue death and leaky blood vessels. Notably, significant deficits showed up after day 82. These findings showed the importance of taking into account blood–brain barrier (BBB) disruption, vascular remodeling and delayed radionecrosis when investigating the effects of radiation in preclinical models [38].

### 3.3. Modulation of Radiation-Induced Neural Damage and Recovery

Environmental enrichment also partially restores hippocampal neurogenesis following high-dose irradiation independent of blood monocyte–derived microglia. Ruitenberg and colleagues investigated whether delayed exposure to environmental enrichment or voluntary exercise can rescue adult hippocampal neurogenesis after high-dose (10 Gy) total body irradiation in mice [39]. A bone marrow chimera model that tracked peripheral monocyte-derived microglia offered the authors their best evidence to date for the dramatic and persistent inhibition of progenitor cell proliferation and neurogenesis in the dentate gyrus under sedentary conditions. Importantly, eight weeks of stimulation of the granule cell layer by environmental enrichment remarkably increased the number of BrdU- and doublecortin-positive cells, which represents recovery of the neurogenic process. Microglial density was significantly increased in the dentate gyrus. Most crucially, this increase was due to local proliferation of resident microglia, as opposed to an influx of circulating CX3CR1^+^ monocytes. These findings indicate that a population of latent neural precursor cells survives even after severe irradiation and can be reactivated by environmental enrichment through mechanisms independent of blood monocytes and derived microglia [39].

In addition, it is suggested that autophagy modulates radiation damage in the developing brain in a key way. Wang and colleagues showed that selective blocking of autophagy by neuronal deletion of *Atg7*, a major autophagy gene, significantly attenuated irradiated neural stem and progenitor cell death in juvenile mice [40]. Postnatal day 10 *Atg7* deficient mice that were irradiated at the whole brain level, greatly inhibited apoptotic cell death in the dentate gyrus and the cerebellum without affecting baseline neural progenitor proliferation. Inhibition of autophagy also minimized microglial activation and attenuated the radiation-induced inflammation, evidenced by decreased proinflammatory cytokines and chemokines levels especially in the cerebellum. These neuroprotective advantages were associated with modifications in CX3CL1–CX3CR1 signaling, establishing the correlation between neuronal autophagy and microglial activation, as well as neuroinflammation. Together, these data showed autophagy at the core of radiation-induced neurotoxicity in the immature brain and implicated autophagy inhibition as a potential therapeutic strategy to counter long-term cognitive and developmental side effects following pediatric cranial radiotherapy [40].

### 3.4. Synergistic Effects of Radiomodulation for the Treatment of Brain Tumors

Recent studies are now linking the effects of radiomodulation combined with treatments for brain tumors. Non-ablative low doses of IR delivered to small animal intracranial models of glioma (GBM) brain tumors combined with focused ultrasound-mediated blood–brain barrier disruption (FUS-BBB), can modulate the neurovascular tumor microenvironment (TME) to enhance delivery of therapies in preclinical glioma models [41]. In GBM containing mice, the combination of 2 Gy RT and FUS-BBB significantly prolonged survival compared to 2 Gy RT alone, though not compared with single-dose 5 Gy irradiation. Gene expression analysis showed extensive alterations in several biological networks, including a strong upregulation of genes related to angiogenesis (ANGPT), immune regulation (TGFβ, Galectin), inflammation (CCL/CXCL), cell growth and differentiation (FGF, BMP, IGF), vascular stability (EDN), metabolic control (RBP4), and neurotrophic factors (BDNF, PTN). These results suggest that radiomodulation alters the neurovascular and TME, suggesting new avenues for combination treatments in translational neuro-oncology research [41].

Zeng and colleagues reported additive survival effects in GBM mice treated with immunotherapy and SRS, compared to mice treated with monotherapy [42]. At the tissue level, combination therapy decreased the infiltration of CD4^+^/FOXP3^+^ regulatory T cells and increased the recruitment of CD8^+^/IFN-γ^+^/TNF-α^+^ cytotoxic T cells, suggesting a pro-inflammatory TME, supporting the ability of non-ablative doses of IR to modulate the TME as a means of improving outcomes when combined with multimodal therapies for the treatment of gliomas [42]. Taken together, radiomodulation can play a versatile role in the treatment of a multitude of neurological conditions, summarized for our readers in Table 1.

## 4. Use of SRS for the Treatment of Functional Disorders

SRS delivers therapeutic radiation to deep brain targets with submillimeter spatial accuracy, enabling intervention within eloquent regions [53]. Beyond its geometric precision, SRS seamlessly integrates with modern connectomic and functional connectivity workflows, allowing clinicians to execute network-informed therapy that is individualized to each patient’s neuro-architecture [54]. Importantly, the biological effects of SRS are increasingly recognized to extend beyond focal tissue ablation, encompassing a spectrum of neuromodulatory effects that can be leveraged to refine therapeutic outcomes; this parameter space makes SRS a highly attractive therapy for optimizing rehabilitation of complex neural dynamics non-invasively [10,55].

Though SRS was initially conceptualized and employed by Leksell to create lesions via radiation-induced free radical formation and cell death, decades of research demonstrate that meaningful and durable changes in circuit physiology occur below histologic thresholds for frank necrosis [11,56]. Though work continues to elucidate underlying biological mechanisms, emerging evidence suggests that SRS exerts subnecrotic effects via selective modulation of cells with high turnover rates, particularly cycling glial cells, with relative preservation of non-dividing neurons. Beyond this lies a broader neuromodulatory zone, in which subtle biochemical, inflammatory, and microstructural changes occur without significant cell death. Inflammatory mediators produced within the subnecrotic region are thought to contribute to functional changes in the surrounding neuromodulatory area, while regions outside this zone remain unaffected [57]. The relative extent of each zone depends not only on delivered dose but also on treatment volume, tissue composition, vascular sensitivity, and individual genetic susceptibility. White matter tracts and microvasculature are particularly radiosensitive, with diffusion-weighted MRI demonstrating vasogenic and cellular edema patterns consistent with fiber dissociation and myelin sheath alteration rather than axonal loss [57]. This neuromodulation framework substantially expands the therapeutic scope of SRS beyond its initial conception as an ablative technology. In the following sections, we highlight how these neuromodulatory properties underpin the clinical utility of SRS across pain, epilepsy, and other functional neurological and psychiatric disorders.

### 4.1. SRS for the Treatment of Epilepsy

Over the past two decades, the utility of SRS-mediated neuromodulation in epilepsy has continued to expand. As epilepsy is increasingly conceptualized as a network disorder—arising from aberrant seizure onset zones, propagation pathways, and thalamocortical loops—neuromodulation has become a cornerstone of treatment for patients who are poor candidates for resection. Modalities such as vagus nerve stimulation, responsive neurostimulation, and anterior thalamic DBS exemplify this shift by reshaping network excitability and synchrony without tissue destruction [58,59,60].

In mesial temporal lobe epilepsy (MTLE), randomized and prospective studies demonstrate that SRS offers seizure remission rates comparable to open surgery in selected patients, albeit with delayed onset. A study of 15 MTLE patients by Bartolomei and colleagues reported seizure cessation in 60% of patients with a mean follow-up of 8 years, with seizure cessation occurring with a mean delay of 12 months after GK SRS [61]. Similarly, a prospective multicenter study by Regis and colleagues assessing the efficacy and safety of GK SRS for the treatment of medically refractory epilepsy (MRE) patients reported a reduction in median seizure frequency from 6.16 prior to treatment to 0.33 at 2 years after treatment [62]. The ROSE trial randomized MRE patients between open surgery and SRS, demonstrating seizure remission in 52% of patients who were treated with SRS at 2 years follow-up, increasing up to 74% after 3 years [63]. Adverse effects are predominantly delayed and radiobiologic, including transient edema and inflammatory changes, with lower immediate cognitive morbidity than dominant-hemisphere resection [63].

SRS has also proven effective in hypothalamic hamartoma–related epilepsy, a particularly challenging condition. A recent meta-analysis of 152 patients reported seizure improvement in 77% and seizure freedom in 48%, with an adverse radiation effect rate of 0% [64]. Similarly, Romanelli and colleagues reported their experience using image-guided LINAC radiosurgery to treat hypothalamic hamartomas, demonstrating complete seizure freedom in eight patients with no neurological complications reported that are associated with microsurgical resection [65].

The level II evidence provided by the 2018 ROSE randomized trial currently represents the strongest evidence supporting SRS for the treatment of epilepsy; however, SRS remains less efficient than anterior temporal lobectomy (ATL) at achieving post-treatment seizure remission. This study had a small recruitment size and an early study termination at 34–36 months, preventing comparison of longer-term seizure remission between the two treatment modalities [63]. The 2008 level IV cohort study by Bartolomei and colleagues of 15 patients treated with Gamma Knife SRS for MTLE with greater than 5 years of follow-up reported a mean delay of seizure cessation of 12 months, with 60% of patients requiring a short period of steroid treatment for mild headaches, and all patients eventually experiencing relapse requiring restoration of antiepileptic drug therapy [61]. Given that these small powered studies are now between a decade to two decades old, it behooves the field to properly design a multicenter randomized clinical trial with careful patient selection and standardization of treatment dose and target to confidently conclude the safety and efficacy of using SRS to treat medication-refractory epilepsy.

### 4.2. SRS for the Treatment of Essential Tremor

Essential Tremor (ET) is one of the most common adult movement disorders, characterized by an isolated upper limb action tremor with at least a 3-year duration, although other symptoms may occur [66]. A neurodegenerative disease, MRI studies show changes in metabolism and relations to the cerebellum and motor cortex activity. ET per se is functionally disabling, but complications of ET can bring on additional symptoms that complex behavior [66]. Common comorbidities in patients include hypertension (52%), other nervous system disorders (45%), lipid metabolism disorders (44%) and mood and anxiety disorders (37%). Hearing impairment, sleep disorders, and psychiatric disturbances are also comorbidities that follow ET conditions, emphasizing the need for a reliable and effective treatment [66].

Evidence supporting the use of SRS to treat ET is weak. Of the 34 studies included in Martinez-Moreno and colleagues’ systematic review, 14 had fewer than 10 patients, and 8 were case reports [67]. Treatment was usually lesion-based Gamma Knife thalamotomy using a single 4 mm collimator, with doses ranging from 100 to 200 Gy. The mean improvement in tremors was 88%. While degrees of improvement were not closely monitored in many studies, those that kept records reported a 31% rate of tremor disappearance and various positive responses. Similar to the epilepsy literature, ET studies reported a delayed post-treatment response, with a mean of 4.8 months and a median of 2.5 months. Compared to deep brain stimulation, SRS lacks the ability to confirm the target’s location via intraoperative monitoring, thereby increasing the need for highly accurate anatomical identification during SRS treatment planning. Targeting the ventral intermediate thalamus (VIM) using ablative doses of SRS requires precise MRI-guided volumetric dose and treatment planning [66,67,68] but remains inferior in its inability to achieve closed-loop feedback unlike deep brain stimulation (DBS). Conversely, thalamotomy harbors a high rate of morbidity compared to SRS and has largely fallen out of favor [67]. Gamma Knife, CyberKnife Robotic Radiosurgery, and Zap-X Gyroscopic Radiosurgery (Zap-X) are currently the three SRS devices in use across the world [68]. These platforms are largely similar in safety and efficacy; however, as the Zap-X has the smallest collimator range (3–4 mm), the smallest focus may be more advantageous in treating tremors with a lower side effect profile [68]. Complications such as dysarthria and disequilibrium may occur, compared to DBS-related complications, which are quite low [66]. With the increasing availability of SRS devices across the world and the United States, the non-invasive and relative low cost of this treatment modality compared to other more expensive ablative treatment modalities such as magnetic resonance-guided laser interstitial thermal therapy, radiofrequency thermocoagulation, and focused ultrasound, allows SRS to serve as a bridge for MRE patients who are not ideal surgical candidates. For the convenience of our readers, we have provided a summary table of studies using SRS for the treatment of epilepsy and tremors (Table 2).

## 5. Use of SRS for the Treatment of Pain Disorders

### 5.1. SRS for the Treatment of Trigeminal Neuralgia

Trigeminal neuralgia (TN) is a clinically diagnosed neuropathic disorder characterized by recurrent, unilateral, brief, electric shock-like pains affecting one or more divisions of the trigeminal nerve, often triggered by innocuous stimuli such as touch, chewing, or speaking [69]. Traditional therapy consists of anticonvulsant medications, with microvascular decompression representing the preferred surgical option for eligible patients. Percutaneous ablative procedures, including radiofrequency rhizotomy, glycerol rhizolysis, and balloon compression, as well as stereotactic radiosurgery, are typically reserved for recurrent disease or patients who are poor surgical candidates.

The clinical utility of SRS is supported by the robust outcomes literature demonstrating safety and efficacy in drug-resistant TN. A systematic review reported initial pain-free outcomes ranging from approximately 17–100% depending on treatment platform (Gamma Knife [mean 53.1%, median 52.1%], LINAC [mean 49.3%, median 43.2%], CyberKnife [mean 56.3%, median 58%]), with durable benefit observed in many patients 12 months post procedure [70]. Large historical cohorts encompassing over 400 patients further demonstrate high rates (>90%) of pain control at 12 months following GKS, and long-term single-institution series have reported that >75% of patients remain pain free or experience meaningful improvement 3 years after treatment [71,72].

Although SRS for TN is often framed as an ablative intervention targeting the trigeminal root entry zone, it serves as an example of how neuromodulation and microstructural injury can coexist. Though classical explanations attribute pain relief to radiation-induced micro-injury that disrupts aberrant ectopic firing and ephaptic transmission, emerging evidence suggests a complementary glial mechanism. Within this mechanism, Somaza and Montilla propose that radiosurgery may also alter the function of satellite glial cells within the trigeminal (Gasserian) ganglion, which play a critical role in regulating neuronal excitability. Modulation of these glial elements may further suppress pathological firing and pain signaling, compounding the therapeutic effect [73,74]. Radiosurgery has been used to treat TN since the 1960s with an average of 21.5% of patients reporting post-treatment hypesthesia, dry eye syndrome, and jaw weakness. Several studies have reported long-term outcomes after radiosurgery, with a total of 920 patients. Among them, Dhople and colleagues reported a median follow-up of 5.6 years, an initial pain-free rate of 81%, and a gradually decreasing percentage who maintained pain relief over time [75]. Kondziolka et al. followed 107 patients for more than 5 years and reported higher pain-free rates initially and over time [76]. Unlike treatment of SRS for epilepsy and tremors, SRS for TN leads to a rapid treatment response, likely due to direct lesional effects on the sensory nerve fibers of the cisternal segment of the trigeminal nerve, and represents a safe and effective modality of the treatment of TN.

### 5.2. Central Neuropathic Pain and Brain Targets

SRS for the treatment of central neuropathic pain targets the medial thalamus (intralaminar CM/Pf ± mediodorsal nuclei), the central lateral thalamus (CL/CLp), and the anterior cingulate cortex [77,78]. In a 56-patient single-group experience, radiosurgery to the medial thalamus—alone or combined with trigeminal or pituitary targets—achieved ≥50% pain relief in approximately 69% of patients [79]. A systematic review encompassing six studies and 125 patients reported meaningful pain reduction in approximately 55%, persisting in 38% at last follow-up, with low rates of morbidity [80]. Clinical benefit is typically delayed, with median onset around three months, and eventual recurrence of symptoms [78,81]. In a cohort study of 21 patients, Gamma Knife CL thalamotomy resulted in meaningful pain reduction in 57% of patients after a median of three months, with declining durability over time [78]. This series supports the feasibility and safety of SRS thalamotomy.

Anterior cingulotomy using SRS has the purported mechanism of modulating the affective–motivational dimension of pain rather than decreasing pain sensation alone, suggesting that it may be used to treat patients with diffuse chronic pain with a prominent psychological component. A small series of 5 patients reported a 60% improvement in symptoms [82]. With a median follow-up of 24 months across a range of radiation doses, pain relief was reported in only 43.3% of patients. As such, the jury is still out as to whether or not there is a role for SRS in the treatment of central neuropathic pain. We have provided a summary table for our readers detailing the use of SRS for the treatment of different pain conditions (Table 3).

## 6. Use of SRS for the Treatment of Psychiatric Disorders

### 6.1. SRS for the Treatment of Obsessive–Compulsive Disorder

Refractory obsessive–compulsive disorder (OCD) has been treated in 14 Gamma Knife (GK) SRS capsulotomy clinical studies, a dataset comprising 142 resistant OCD patients [83]. Patients reported improvement in their symptom severity using the 10-item, clinician-administered Y-BOCS scale in approximately 96% of studies, with doses ranging from 60 Gy to 90 Gy delivered to the anterior limb of the internal capsule, and with variation across bilateral, double-shot GK SRS capsulotomies. A large number of patients (78 total) received 90 Gy of irradiation, with varying Y-BOCS outcomes ranging from remission to remaining within the “extreme” range. Doses below 90 Gy have been implicated in achieving similar or even better effects. The most adverse effects were mild, ranging from nausea, vomiting, and headaches. However, brain cyst development has been reported as a potential complication of GK SRS and was observed in 10% of patients in another study using a higher dose range (160–200 Gy) [84]. While the application of Gamma Knife capsulotomy for the management of treatment-refractory obsessive–compulsive disorder is clinically effective, the use of such doses (60–90 Gy at the anterior limb of the internal capsule) is better understood as lesions than as definitively non-lesional forms of radiomodulation. It is worth noting that the former is an accepted but high-dose form of radiosurgery-based lesioning that is known to be effective and must be distinguished from the new paradigm of radiomodulation discussed earlier in the text. It remains an open question of whether lower, subnecrotic doses could achieve comparable benefit through genuine neuromodulation of capsular circuitry.

### 6.2. SRS for the Treatment of Alcohol Use Disorder

The promise of using SRS to treat addiction comes from recent preclinical studies using a miniature pig model of alcohol use disorder (AUD) [85]. Yeh and colleagues recently reported the ability to safely deliver low-dose (Dmax < 40 Gy, 7.5 mm collimator) focal IR to the brains of miniature pigs without adverse neurological sequelae or evidence of ablative neuronal tissue injury [52]. They exposed Lee Sung miniature pigs to ethyl alcohol in amounts exceeding the human binge drinking level over a period of two years to develop alcohol dependence. These animals then underwent single-fraction bilateral SRS of the nucleus accumbens (NAc; Dmax = 30 Gy, 5 mm collimator) and their voluntary alcohol consumption was measured over a period of 1 year. Brain MRIs were repeated every 3 months to assess radiation-induced tissue changes in the NAc. Voluntary alcohol consumption was significantly decreased for up to 9 months post radiosurgery [85]. Post-irradiation effects consisted of one animal that was seen to have a significant structural alteration in the form of an asymptomatically enlarged lateral ventricle, which was noted to imply collateral effects. While encouraging, the findings remain limited to a mini-pig model. Currently, there is no published data on the use of SRS in humans for treating AUD, and thus further research needs to be conducted before translating this approach into a clinical setting.

### 6.3. SRS for the Treatment of Bipolar Depression

Refractory Bipolar Depression has been treated in a small-scale clinical study in which three screened volunteers were subjected to bilateral subgenual cingulate cortex (SGC) irradiation with a dose of 75 Gy in one fraction, and observed over a 1-year period [86]. Two of the three volunteers displayed clinical improvement in their depressive symptoms and had a mean decrease in HDRS-17 of 27% following their initial and post-treatment follow-up. The remaining one had no change in severity. Post-irradiation, one patient showed manifestation of edema 12 months after, while another patient had edema at the SGC following a long-term follow-up. No other adverse effects have been observed. These results must be interpreted cautiously. The limitation associated with these findings, which includes having a sample size of just three individuals, no control group, limited response rate (only two of three), and radiation-induced edema in two subjects, means that no conclusion about clinical readiness can be made at this point. These results are only meant to demonstrate the feasibility of the procedure, and no determination about its clinical readiness can be made based on these findings. Larger-scale randomized clinical trials will need to be conducted to prove clinical efficacy.

The aforementioned three psychiatric applications vary in the degree of available supportive evidence and in the extent of their congruence with the discussed non-ablative radiomodulation paradigm, all failing to satisfy both of these requirements. Gamma ventral capsulotomy for OCD benefits from the greatest body of scientific literature, but the former consists of an aggregate of 14 different studies (n = 142) that involve variability in dosage and technique used as well as diverse outcomes even at a fixed dose, ranging from remission to constant presence of severe symptoms. Even more importantly, it is delivered in a dosage range of 60 to 90 Gy and should rather be referred to, as it was already indicated above, as a well-established lesioning procedure and not as a non-ablative modulation approach. Therefore, despite being the most evidenced and supported among the applications under discussion, it can hardly be called a representative of the paradigm. The literature on OCD includes an example of an opposing position regarding the need for lesioning: even though most patients received 90 Gy, lower dosages were also reported to provide either equally good or even better results, thus raising the issue of whether it is possible to receive comparable therapeutic effects through non-ablative neuromodulation of the circuits involved. Of the above three psychiatric conditions, gamma ventral capsulotomy for OCD is by far the most established technique. SRS for the treatment of AUD and bipolar depression is still in its infancy and will require rigorous clinical trials testing to demonstrate safety and efficacy compared to current standard of care practices. Finally, refractory bipolar depression is the least developed of the three: an uncontrolled three-patient feasibility study, which included one non-responder and involved the occurrence of radiation-induced edema at the same frequency as clinical improvement (two out of three patients), while its authors refer to it as an illustration of feasibility rather than efficacy. For ease of reference for our readers, we have provided Table 4 summarizing our critical appraisal of the current literature on the use of SRS for the treatment of psychiatric disorders.

## 7. Discussion

There is currently a lack of longitudinal follow-up data regarding the longer-term effects of SRS-induced neuromodulation in humans. As suggested by Regis and colleagues in 2004, “…further basic science work is required to better understand the effects of dose, volume, target topography, and dose distribution homogeneity on the modulation of specific biological systems” [62]. Zaer and colleagues recently published an elegant study in Göttingen minipigs in which they treated their visual cortex with a 7.5 mm-diameter target using 40, 60, 80, and 100 Gy of SRS [57]. A 9 mm-wide, 8 shank, 64-electrode probe was implanted into their brains spanning the radiation focus and neighboring low-exposure neighboring areas 6 months post-irradiation to assess neuronal network function. Unirradiated animals subjected to visual stimuli resulting in typical visual evoked potentials (VEPs), while animals irradiated with 40 Gy IR demonstrated a significant reduction in the P1 peak time, indicating a higher network excitability, while doses of 60 Gy were associated with a decrease. Post-mortem histology demonstrated no evidence of necrosis at doses below 60 Gy. They then co-cultured inducible pleuripotent human stem cell-derived neurons and astrocytes in vitro and exposed them to IR, and performed electrophysiologic recording of excitatory and inhibitory neurons, revealing a higher susceptibility of inhibitory over excitatory neurons to the effects of IR [57]. The significance of this study demonstrated a long-lasting IR dose-dependent neuromodulation effect. Similarly, a single focal irradiation of the bilateral NAc in a miniature pig model of alcohol dependency achieved a durable reduction in voluntary alcohol consumption up to 9 months [52], further supporting the ability of sublethal doses of IR delivered to specific relay centers in the brain to modulate neural function. We eagerly await further in-human studies to see whether these preclinical findings will translate.

The potential for using neuromodulation for the treatment of psychiatric illnesses such as AUD, substance dependencies, and treatment-resistant depression, comes from reports of improved alcohol abstinence in patients [87,88,89,90,91]; decreased opiate, benzodiazepine, and methamphetamine dependencies [92,93,94,95,96,97]; and decreased depressive symptoms [98,99,100]. However, significant ethical concerns exist arising from past experiences of using SRS to treat patients with alcohol dependency, which brings into question the appropriateness of using this modality to treat this patient population. These ethical concerns will be outlined below in Section 8.

## 8. Limitations

Due to the non-invasive nature of SRS, unlike neuromodulation through implantation of DBS electrodes, allowing for real-time neurophysiologic feedback of the stimulatory response, we currently do not have the ability to perform real-time feedback of the neuromodulatory effects of SRS. We also currently do not know how often SRS of the NAc can be repeated for the treatment of AUD and other psychiatric illnesses to achieve a life-long steady state of wellness, and the cellular and molecular effects of neurotransmitter receptor signaling following repeated SRS treatments. Schneider and colleagues compared the duration of the neuromodulatory effects of different neuromodulation modalities and compared them with radiomodulation, postulating that while radiomodulation may potentially achieve permanent durable effects, it is limited to unidirectional, downward neuromodulation, unlike the bidirectional capabilities of electrical cortical stimulation, DBS, transcranial magnetic stimulation, and optogenetic stimulation [13].

Another significant limitation with SRS is the delayed period of time before a therapeutic effect is observed (several months to a year), corresponding to the initial period of radiation-induced axonal degeneration and neuronal dysfunction, mandating that the patients remain on their pre-existing treatment regimens with close follow-up [101]. Furthermore, the side effects of SRS can be varied, ranging from a self-limiting headache to weight gain and memory loss. In addition, significant ethical considerations exist in prescribing SRS for the treatment of psychiatric disorders. The NAc is a complex relay hub that processes human motivation, learning, and emotions. Ablating the NAc risks decoupling the brain’s ability to perform “human” functions, form positive reinforcement-based memories, sustain natural motivation for essential work day-to-day activities, and maintain hedonic capacity [102]. Large-scale treatment of patients in addiction centers across China in the early 2000s using NAc radiofrequency ablation purported temporary curbing in dependence. However, they were left with severe and lasting iatrogenic adverse effects, leading to a country-wide ban on the procedure by 2004 [103]. As such, we must exercise an abundance of caution before using SRS for the treatment of psychiatric and functional disorders. Stringent basic science and preclinical studies must be performed to better understand the downstream effects of SRS in order to reach an acceptable risk-benefit profile for patients [104,105,106].

## 9. Future Directions

More recently, investigators have begun to use connectomic-guided SRS by integrating functional MRI (fMRI), MRI, and diffusion brain imaging data from the FSL-FMRIB software library tools (https://fsl.fmrib.ox.ac.uk/fsl/docs/ accessed on 22 April 2026) and a 12 million-fiber whole-brain tractography template to generate fiber density maps compared against the XTRACT Human Connectome Project Probabilistic Tract Atlas. Using connectivity planning in the Brainlab Elements intraoperative neuronavigation suite (https://www.brainlab.com), Lovo and colleagues mapped pain-processing nuclei in patients’ brains during SRS-guided thalamotomy procedures for the treatment of chronic pain [54]. Connectivity analysis demonstrated that fiber tracts within the contoured SRS treatment plan extended upward to the primary motor and sensory cortices while descending tracts reached the periaqueductal gray and critical pain processing centers of the prefrontal cortex, insula, amygdala, and cerebellum. This study demonstrated that integrating connectivity mapping during SRS could be a feasible means of adopting a personalized approach to achieving neuromodulation using SRS. Similarly, Chang and colleagues from the Lovo group used connectomics to map the NAc using DTI for GK SRS planning in 5 patients [107]. Middlebrooks and colleagues recently performed a retrospective study of patients in a prospective trial for frameless virtual-cone SRS on a LINAC for Essential Tremor or tremor-dominant Parkinson’s Disease aimed at developing a new targeting approach by using patient-specific structural connectivity to improve outcomes after SRS [108]. Using connectomic-based targeting and probabilistic tractography assessed connectivity from each thalamic voxel to the primary motor cortex, primary sensory cortex, and supplementary moto area/premotor cortex. Results of the study showed that patient-specific connectivity between the treatment target and the primary motor cortex positively correlated with treatment outcomes, providing a practical targeting method for SRS thalamotomy for tremors.

Finally, a study by Zaer and colleagues describes an intracortical implantable brain–computer interface (BCI) for telemetric real-time recording and manipulation of neuronal circuits in the visual cortex of Göttingen minipigs after exposure to IR [109]. This device allows for untethered BCI telemetric recording of local signatures of sub and suprathreshold neuronal activity in the visual cortex with high bandwidth, capable of long-term untethered real-time communication for causal preclinical circuit-based closed-loop interventions. This untethered implantable BCI has widespread functionality and can facilitate the study of the neuromodulatory effects of sublethal doses of IR delivered to different parts of the brain in preclinical animal models, serving as a powerful tool for understanding downstream effects on neuronal signaling and feedback across circuits following exposure of the brain to sublethal doses of radiation. This preclinical data set can help inform clinicians to design future clinical trials to determine the safety and treatment parameters for using SRS to treat patients with functional and psychiatric illnesses.

## 10. Conclusions

In conclusion, there is a growing body of preclinical data that describes the ability of varying doses of focused radiation to achieve radiomodulatory effects in the brain, with ongoing work to determine if this phenomenon can be leveraged for clinical treatment applications. There are ongoing ethical concerns for causing long-term, irreversible neurologic and psychiatric dysfunction in patients following ablation of the NAc for the treatment of their psychiatric disorders. Leveraging these preclinical animal models to study the radiomodulatory effects of varying doses of focused radiation to the NAc and other nuclei may help to inform clinicians in the design of future clinical trials to improve the safety profile of using SRS for the treatment of functional and psychiatric disorders. Finally, the emerging use of connectomics to assist in the accurate identification of anatomical nuclei during radiosurgery planning can help improve the safety and treatment efficacy for patients. In closing, Dr. Leksell first described the phenomenon of radiomodulation in the 1950s. With the renewed interest in radiomodulation research across the world, perhaps we should rework the title of this review to *“Radiomodulation—Back To the Future”*.

## Figures and Tables

**Table 1:** Summary of preclinical studies of radiomodulation. Summary of preclinical rodent, swine, and squid laboratory models used to study cellular responses following SRS treatments to the brain.

Study	Species/ Strain	Experimental Model	Radiation Target	Radiation Modality	Dose/ Fraction-ation	Sample Size (n)	Follow-Up Duration	Primary Endpoints	Key Findings	Main Limitations
Mullin et al., 1986 [30]	Male Sprague-Dawley rats	Brain synaptosomes	Brain synaptosomes	High-energy electrons, γ-rays	100–10,000 rad	4–6 experiments	Seconds–minutes	^22^Na^+^ uptake, STX binding, membrane fluidity	Dose-dependent inhibition of sodium channel function without changes in membrane fluidity or STX binding.	In vitro model only. Acute endpoints only. No behavioral or histological outcomes. No long-term assessment. Mechanism not fully defined.
Tolliver & Pellmar, 1987 [27]	Male guinea pigs	In vitro hippocampal slices	CA1 hippocampal region	^60^Co γ-radiation	6.25–200 Gy; 5 or 20 Gy/min	4–17 per dose group	≤1.5 h post-irradiation	Population spike (PS), population synaptic potential (pop PSP), afferent volley, neuronal excitability	Dose- and dose-rate-dependent synaptic impairment. Postsynaptic damage at ≥75 Gy. Higher dose rates enhanced synaptic damage.	In vitro model. Acute assessment only. No behavioral outcomes. No histological analysis. Single brain region (CA1).
Pellmar, Schauer & Zeman, 1990 [33]	Male guinea pigs	In vitro hippocampal slice preparation	Hippocampal slices (CA1 region)	17.4 keV X-rays	5–65 Gy; 1.54 Gy/min	Control (n = 11); 5 Gy (n = 7); 10 Gy (n = 8); 20 Gy (n = 10); 30 Gy (n = 6); 40 Gy (n = 6); 50 Gy (n = 6); 65 Gy (n = 6)	Electrophy-siological recordings before, during, and up to ~60 min after irradiation.	Synaptic efficacy (afferent volley vs. pop PSP). Spike generation (pop PSP vs. population spike). Input-output curves.	Doses ≥40 Gy increased synaptic efficacy and reduced spike generation. Damage progressed after irradiation. No recovery observed during the observation period.	In vitro model. Acute assessment only. No behavioral outcomes. No histological analysis. Single brain region (CA1).
Yamagu-chi, Yamas et al. 1991 [28]	Mongrel dogs (8–12 kg)	In vivo canine model of delayed cerebral radiation necrosis	Left cerebral hemisphere	X-ray irradiation (10 MeV linear accelerator)	Single dose 15 Gy	25 dogs (19 irradiated, 6 controls)	3, 6, 9, 12, 15, and 30 months post-irradiation	Light microscopy, electron microscopy, flow cytometry of endothelial cells, Feulgen hydrolysis/DNA analysis	Spongy degeneration appeared at 6 months. Necrotic foci developed at 9–15 months. Endothelial hyperplasia, proliferation, luminal narrowing, increased pinocytosis, and altered DNA kinetics observed. Endothelial cell proliferation peaked at 9 months.	Single-dose design. Canine model. Endothelial-focused analysis. No functional or behavioral outcomes. Limited mechanistic assessment.
Pellmar & Lepinski, 1993 [32]	Male Hartley guinea pigs	In vivo whole-body irradiation followed by ex vivo hippocampal slice electrophysiology	Hippocampus (CA1 region)	^60^Co γ-radiation	5 Gy (1 or 20 Gy/min); 10 Gy (20 Gy/min)	≥6 animals per experimental group	30 min, 1 day, 3 days, and 5 days post-irradiation	Synaptic efficacy, spike generation, population spike input-output relationships.	5–10 Gy γ-radiation produced dose-, dose-rate-, and time-dependent neuronal dysfunction. Early and persistent alterations in synaptic transmission and spike generation. Incomplete recovery after higher-dose exposure.	In vivo animal model. Short follow-up (5 days). No behavioral outcomes. No histological analysis. Mechanisms not directly investigated.
Brisman et al., 2003 [43]	Male Sprague-Dawley rats (250–350 g)	In vivo unilateral hippocampal proton beam radiosurgery model	Right hippocampus	Proton Beam Radiosurgery (PBR)	5–130 CGE, single-fraction	41 irradiated rats, 3 controls	3 months post-irradiation	Morris Water Maze, MRI, electrophysiology, histology, immunohistochemistry	≥90 CGE caused necrosis, cognitive deficits, MRI abnormalities, and impaired hippocampal function	Single time point; small group sizes; normal animals only.
Jenrow et al., 2004 [44]	Adult male Wistar rats	Amygdala-kindling epilepsy model	Basolateral amygdala	Stereotactic ^60^Co- gamma irradiation	18 Gy or 25 Gy, single fraction	Histological analysis: n = 3/group	~6 months	Neuronal density, PV-positive interneurons, histology	Prevention of kindling-induced hippocampal neuronal loss at 25 Gy; no radiation necrosis observed.	Small sample size; single follow-up time point; no mechanistic analysis.
Ben Abdallah et al., 2007 [45]	Adult C57BL/6J mice	Normal brain irradiation model	Whole brain cranial irradia-tion	X-ray irradia- tion	Single dose 4 Gy (27.5 cGy/min)	48 mice (24 male, 24 female)	16 h, 48 h, 1 week, 1 month	Hippocampal cell proliferation (Ki67), neurogenesis (DCX, NeuroD), cell death, granule cell number	4 Gy caused an acute ~80% reduction in hippocampal proliferation and a 60–70% reduction in immature neurons, but both recovered to baseline within 1 month; no loss of mature granule cells was observed.	Low-dose normal-brain model only; no behavioral or cognitive testing; short follow-up limited assessment of long-term effects.
Vlkolinsky et al., 2008 [36]	Male C57BL/6 mice	In vivo heavy-ion radiation model with ex vivo hippocampal electrophysiology	Whole brain	^56^Fe-particle irradiation (high-LET heavy ions)	1, 2, or 4 Gy, single exposure	n = 8–16 per group (dose- and time-dependent cohorts)	1, 3, 6, and 12 months	Hippocampal LTP, population spike amplitude, synaptic efficacy, response to LPS challenge	High-dose irradiation (4 Gy) caused long-term hippocampal dysfunction and impaired synaptic activity.	No behavioral assessment; limited histopathological correlation.
Zeng et al., 2013 [42]	Female C57BL/6 mice	Orthotopic intracranial GL261-Luc glioma model	Intracranial glioma (left striatum)	Stereotactic radiosurgery (SARRP)	10 Gy, single fraction	6–9 mice/group	Up to >180 days	Overall survival, immune response, tumor rechallenge	RT + anti-PD-1 induced long-term survival and durable antitumor immunity.	Single murine glioma model; limited clinical generalizability.
Zou et al., 2013 [46]	Female WT, EC-SOD knockout, and EC-SOD-overexpressing C57BL/6J mice	Normal brain irradiation model	Whole brain (cranial irradiation)	Gamma irradiation	5 Gy, single fraction	n = 5–12 per genotype/treatment group	1–7 weeks post-irradiation	Hippocampal neurogenesis (BrdU, Dcx, NeuN), microvasculature (CD31), neurogenesis-related gene expression	5 Gy reduced hippocampal neurogenesis; EC-SOD overexpression preserved neurogenesis after irradiation.	No behavioral assessment; short follow-up duration.
Cheng et al., 2016 [47]	Male C57 mice (2, 4, 6, 12, and 18 months old)	Aging and hippocampal irradiation model	Hippocampus (dentate gyrus region)	X-ray irradiation	5 Gy, single fraction	≥3 mice per age and treatment group	9 weeks post-irradiation	Hippocampal neurogenesis (BrdU, NeuN, DCX, type 1/type 2 progenitors), microglial activation (Iba1)	Aging reduced neurogenesis, and 5 Gy irradiation caused an additional decline across all ages; irradiation also increased microglial activation.	No behavioral/cognitive outcomes; only male mice studied.
Acharya et al., 2015 [48]	Male WT C57BL/6 and CCR2-knockout mice (2 months old)	Low-dose irradiation and radioadaptive response model	Whole brain (whole-body irradiation) with hippocampal analysis	Gamma irradiation (^137^Cs)	10 cGy, 2 Gy, or 10 cGy + 2 Gy (24 h apart)	64 mice (32 WT, 32 CCR2-KO)	1 month	Hippocampal cell survival (BrdU), neurogenesis (BrdU/NeuN), microglia (Iba1), inflammatory gene expression (CD68, CD11c, Iba1)	Low-dose irradiation reduced hippocampal cell survival and neurogenesis in WT mice, whereas CCR2 deficiency preserved neurogenesis and attenuated inflammatory responses, suggesting a neuroprotective role of CCR2 blockade.	Neurogenesis was assessed by relative cell counts rather than unbiased stereology; no cognitive or behavioral testing.
Constanzo et al., 2017 [38]	Male Fischer rats	Focal brain radionecrosis model	Right S1FL and M1 cortex	Gamma Knife	Single fraction; ~100 Gy maximum target dose	28 rats	140 days	MRI, histology, behavior	MRI detected vascular permeability before overt necrosis; progressive necrosis and later memory impairment developed after irradiation.	Single high-dose model with small MRI cohort and limited intermediate time points.
Cacao & Cucinotta, 2016 [35]	C57BL/6 mice (model based on published mouse datasets)	Mathematical ODE model of radiation-induced hippocampal neurogenesis impairment	Hippocampal dentate gyrus neurogenic compartment (modeled)	Low-LET radiation (X-rays/protons; based on experimental datasets)	Simulated acute and fractionated exposures, mainly 0–10 Gy	No new animals (computational study)	Up to 240 days post-irradiation	Neural stem cells (NSC), neuroblasts (Ki-67), immature neurons (Dcx), apoptosis, microglial activation	Age-dependent model reproduced experimental reductions in neurogenesis after irradiation; neuroblasts were the most radiosensitive population; predicted persistent impairment of hippocampal neurogenesis and better recovery after fractionated versus equivalent acute irradiation.	Computational model relying on previously published datasets and multiple assumptions; limited experimental validation of several parameters.
Wang et al., 2017 [40]	Juvenile Atg7 conditional knockout (Nestin-Cre; Atg7 KO) and wild-type mice	Experimental cranial irradiation model	Whole brain (hippocampal dentate gyrus analyzed)	4 MV X-ray irradiation	6 Gy, single fraction	Histology: n = 7/group; qPCR: n = 5/group	6 h post-irradiation	Apoptosis (cleaved caspase-3, pyknotic cells), proliferation (Ki67, SOX2), microglial activation (Iba1), inflammatory cytokines	Autophagy inhibition (Atg7 deletion) reduced irradiation-induced neural stem/progenitor cell death and neuroinflammation.	Only acute effects were studied (6 h after irradiation); no long-term functional outcomes.
Ruitenberg et al., 2017 [39]	Female Balb/C mice (bone marrow chimeras)	Total-body irradiation followed by environmental enrichment or voluntary running	Whole-body	6 MV photon irradiation (LINAC)	10 Gy total (2 × 5 Gy, 14 h apart)	Sedentary n = 8; EE n = 8; RW n = 6; controls n = 4	16 weeks post-irradiation	Hippocampal neurogenesis (BrdU, DCX), microglia (Iba1, GFP)	Environmental enrichment partially restored hippocampal neurogenesis after irradiation, whereas voluntary running did not.	Small sample size, particularly for DCX analysis in the EE group.
Whoolery et al., 2020 [49]	Male C57BL/6J mice (6 months old)	Whole-body space radiation exposure	^56^Fe and ^28^Si HZE particles	^56^Fe: 20 cGy (fractionated or single dose); ^28^Si: 20 or 100 cGy	Typically 8–12/group depending on experiment	1–4 months post-irradiation	Touchscreen cognitive battery, Location Discrimination (LD), Contextual Discrimination Fear Conditioning (CDFC), PAL, VMCL, PD, PD reversal	Dentate gyrus neurogenesis (DCX+ cell counts)	Space radiation did not impair high-level cognition. Irradiated mice showed significantly improved pattern separation in both appetitive (LD) and aversive (CDFC) tasks despite reduced hippocampal neurogenesis.	Only mature male mice were studied; underlying neural mechanisms of improved pattern separation were not directly investigated.
Cacao et al., 2018 [50]	Multiple rat strains	Mathematical model of hippocampal neurogenesis	Low-LET X-rays/γ-rays	Acute and fractionated exposures	Pooled literature data	Days–months	None	ODE-based neurogenesis model	Acute radiation caused greater neurogenesis impairment than fractionated irradiation; age and strain influenced radiosensitivity.	Modeling study without direct experimental validation or cognitive testing.
Chu et al., 2020 [51]	Adult C57BL/6J mice with or without 9L gliosarcoma	Gliosarcoma brain metastasis model	Brain metastases	SRS	40 Gy	NA	Up to 18 months post-SRS	Tumor response, MRI, behavioral tests, histology	Tumor cleared; cognitive deficits persisted	NA
Yeh et al., 2021 [52]	Lee Sung miniature pigs	Focal radiosurgical neuromodulation model	Left primary motor cortex (M1)	CyberKnife stereotactic radiosurgery	Single dose: 10, 20, 30, 40, 60, 80, 100, or 120 Gy	8 irradiated pigs + 1 control	270 days (9 months)	FDG-PET metabolism (SUV ratio), MRI lesion formation	Doses ≥60 Gy produced persistent reductions in FDG-PET metabolism; MRI-visible lesion occurred only at 120 Gy.	Small sample size (one animal per dose) and no histopathological validation.
Gilly et al., 2021 [34]	*Doryteuthis opalescens* (market squid)	In vivo focal irradiation of squid stellate ganglion	Left stellate ganglion (giant synapse and giant axons)	Focused X-ray irradiation	Single dose: 140–300 Gy	8 irradiated squid	Electrophysiology within 24 h	Synaptic transmission, refractory period, action potential properties	Radiation caused a small but significant shortening of action potential duration and faster repolarization, while synaptic transmission was largely preserved.	Small sample size with limited successful electrophysiological recordings and short follow-up.
Chen et al., 2023 [41]	C57BL/6 mice (preclinical); recurrent malignant glioma patients (clinical)	Preclinical GL261 glioma model and prospective pilot clinical trial	Brain tumor lesions	RT combined with focused ultrasound-mediated BBB opening (RT-FUS)	Mice: RT 2 Gy or 5 Gy; Clinical: re-RT 21–40 Gy (cRT or fSRS) + FUS before RT	Mice: n = 23; Clinical: n = 6	Mice: survival follow-up; Clinical: median PFS 97.5 days	Survival, tumor control, ORR, PFS, safety	RT-FUS improved survival and tumor control versus RT alone in mice; clinically feasible and safe, with ORR 16.7% and no FUS-related adverse events.	Very small clinical cohort (n = 6) with interim analysis and no control group.
Alcazar-Felix et al., 2025 [37]	Female C57BL/6 mice	SRS-induced neuroinflammation and BBB injury model	Left cerebral hemisphere (5 mm target)	Stereotactic radiosurgery (single-fraction X-ray irradiation)	15–60 Gy single fraction	n = 3 per dose and time point	2–18 weeks	BBB disruption, histopathology, neuroinflammation, transcriptomic changes	SRS caused dose-dependent BBB leakage, neuroinflammation, microvascular injury, and activation of microglia, astrocytes, and endothelial stress pathways.	Small sample size, and limited translational applicability to human SRS injury.

**Table 2:** Summary of clinical studies of SRS for functional disorders. This table summarizes studies demonstrating safety and efficacy of SRS for the treatment of functional disorders, including epilepsy and essential tremor. The level of evidence remains weak, largely level III and level IV evidence supporting the current use of SRS for functional disorders. Abbreviations: PT = patient(s); ATL = anterior temporal lobectomy; MTLE = mesial temporal lobe epilepsy; SRS = stereotactic radiosurgery; QOL = quality of life; RCT = randomized controlled trial; FU = follow-up; AED = antiepileptic drugs; HH = hypothalamic hamartoma; GP = globus pallidus; STN = subthalamic nucleus; VL = ventral lateral nucleus; VOA = ventralis oralis anterior nucleus; VOP = ventralis oralis posterior nucleus; UPDRS = Unified Parkinson’s Disease Rating Scale; FTMRS = Fahn–Tolosa–Marin rating scale; VIM = ventral intermediate nucleus; GKRS = Gamma Knife radiosurgery; ARE = adverse radiation effect.

Indication	Study	N	Anatomical Target	Dose	Outcome Measure	Follow Up	Response Rate and Durability	Adverse Events	Level of Evidence
Epilepsy	Barbaro et al., ROSE trial [63]	58 PT total; 31 SRS/27 ATL	Amygdala, anterior 2 cm of hippocampus, and parahippocampal gyrus	24-Gy dose; 50% isodose volume 5.5–7.5 cm^3^. Dose constraints: max 10 Gy to the brainstem and 8 Gy to the optic nerves/chiasm.	Seizure remission/seizure freedom compared with anterior temporal lobectomy	34–36 months; primary endpoint months 25–36	16/31 SRS patients, 52%, achieved seizure remission vs. 21/27 ATL patients, 78% SRS response was delayed; by months 34–36, 23/31 (74%) SRS patients had short-term seizure remission. QOL improved with seizure remission.	SRS: anticipated cerebral edema, transient radiologic/inflammatory changes, headache, transient neurologic deficits, and seizure exacerbation in some patients.	Level II clinical evidence; RCT comparing SRS with ATL for MTLE, limited by early termination resulting in low recruitment, and reduced statistical power (power of 41%)
	Bartolomei et al. [61]	15 PT	Mesial temporal structures: anterior parahippocampal/entorhinal region, hippocampal head/body, and amygdalofugal amygdaloid complex	24 ± 1 Gy marginal dose to 50% isodose; 18 mm collimator; 50% isodose target volume 5500–9000 mm^3^.	Seizure freedom in drug-resistant MTLE lessened AED usage.	Mean ~8 years, range 6–10 years; clinical review and telephone follow-up after year 3	9/15 PT, 60%, were Engel Class I at final follow-up. Seizure cessation occurred after a mean delay of 12 ± 3 months, often preceded by increased auras/seizures in 6/15 PT. Response was generally maintained over long-term follow-up if AED therapy was continued; AED tapering triggered relapse of auras or complex partial seizures in responders.	Mild headache requiring short corticosteroids in 9/15, 60%; visual field changes: hemianopsia in 1, 6%, asymptomatic quadrantanopsia in 8/15, 53%; asymptomatic stable post-GK cyst in 1 PT.	Level IV clinical evidence; long-term single-center case series without a control group.
	Hajikarimloo et al. [64]	7 studies; 152 patients with HH	Hypothalamic hamartoma	Mean prescribed dose ranged 15.5–18.8 Gy; mean maximum dose ranged 21.53–36.8 Gy across studies,	Seizure improvement, seizure-free status, Engel I/II, Engel III/IV; little observed adverse radiation effects	Varies per study. Mean follow-up ranged from 25.5 to 118.2 months across studies	Pooled seizure improvement 77%; pooled seizure-free status 48%; pooled Engel I/II good outcome 67%. Response may be delayed and varied by lesion type, patient factors, and follow-up duration.	Pooled ARE rate 0%; pooled visual impairment 0%; new hormonal deficit 0%, though individual data reported 2/39 new hormonal deficits	Level III clinical evidence; SRS-specific systematic review/meta-analysis of nonrandomized studies.
Essential Tremor	Martínez-Moreno et al. [67]	34 studies; PT number varies by study; single PT to 196 PTs.	Primarily VIM; less commonly GP, VL, VOA/VOP, STN Lesion-based radiosurgical thalamotomy	Usually single 4 mm collimator; dose range 100–200 Gy across studies, with recommended VIM GKRS dose 130–150 Gy	Tremor reduction using variable scales, including UPDRS and FTMRS; quality of life in select studies	Variable across studies; reported follow-up from months to years, with some series reporting up to 228 months.	Mean tremor improvement was approximately 88%; among studies with ≥10 patients, ~82% had clinical improvement. Response was delayed, with mean time to response of ~4.8 months and a median of ~2.5 months. Long-term durability limited by variable/short follow-up.	Complications are rare and often transient; the mean complication rate is ~17%, median 2%. Reported effects: weakness, sensory loss, dysarthria/speech disturbance, dysphagia, edema, and rare hemorrhage.	Level IV clinical evidence; systematic review/practice guideline based primarily on Level IV retrospective series, case reports, and uncontrolled prospective studies. No randomized controlled trials were included.

**Table 3:** Summary of clinical studies of SRS for the treatment of pain disorders. Summarized above are pertinent studies reporting the use of SRS for the treatment of trigeminal neuralgia and chronic neuropathic pain syndrome.

Indication	Study	N	Anatomical Target	Dose	Outcome Measure	Follow Up	Response Rate and Durability	Adverse Events	Level of Evidence
Trigeminal neuralgia	Régis et al. [71]	497 PT	Retrogasserian/cisternal portion of trigeminal nerve	Single 4 mm isocenter; median maximum dose 85 Gy, range 70–90 Gy	BNI pain scale; pain freedom without medication; recurrence; facial hypesthesia	Median 43.8 months, range 12–174.4 months	456/497 PTs, 91.75%, initially pain free, median time 10 days. Pain-free without medication at 3, 5, 7, and 10 years: 71.8%, 64.9%, 59.7%, and 45.3%. Pain relief without further surgery at 10 years was 67.8%.	New sensory dysfunction/hypesthesia occurred in 72 PTs; actuarial hypesthesia rate 20.4% at 5 years and 21.1% at 7 years. Very bothersome facial hypesthesia occurred in 3 patients, 0.6%. No anesthesia dolorosa or dry-eye syndrome reported.	Level IV clinical evidence; large historical cohort with prospectively collected data; non-RCT
Central Neuropathic Pain	Urgosik & Liscak [81]	30 PT with severe refractory pain syndromes	Medial thalamus; centromedian/parafascicular complex	145–150 Gy; single 4 mm collimator shot	Pain relief percentage and BNI pain intensity score	Median 24 months, range 12–180 months	Successful pain relief in 13/30 PT, 43.3%; complete pain relief in 1 PT. Median latency to pain relief was 3 months, range 2–12 months. Pain recurred in 4/13 responders, 31%, after a median of 24 months.	No new neurological deficits, no worsening of existing neurological deficits, and no worsening of pain symptoms reported.	Level IV clinical evidence; retrospective single-center case series without control group.

Abbreviations: PT = patient(s); SRS = stereotactic radiosurgery; QOL = quality of life; RCT = randomized controlled trial; FU = follow-up; BNI = Barrow Neurological Institute; CM/Pf = centromedian/parafascicular complex; GKT = Gamma Knife thalamotomy.

**Table 4:** Summary of Clinical Studies Using SRS for the Treatment of Psychiatric Disorders.

Indication	Study	N	Anatomical Target	Dose	Outcome Measure	Follow-Up	Response Rate & Durability	Adverse Events	Level of Evidence
OCD (Gamma Ventral Capsulotomy)	Camponeschi 2023 (14-study aggregate) [83]	142	Anterior limb of internal capsule (bilateral)	GK SRS; 60–90 Gy; majority 90 Gy bilateral double-shot	Y-BOCS improvement (10-item, clinician-administered)	Variable per study	96% of papers showed improvement on the Y-BOCS scale. The actual range is from remission to sustained severe symptoms. The durability of improvement in responders was reported with variations among papers.	Adverse events described in literature were minor, comprising nausea, vomiting, and headaches. Brain cysts were formed in 10% at the dosage of 160–200 Gy (Peker et al., 2020 [84]).	Level II evidence means systematic reviews of observational cohorts. It should be emphasized that the mode of action here is ablative or lesioning, not radiomodulation.
Alcohol Use Disorder (Preclinical)	Yeh et al., 2025. [85]	N/A (miniature pig model)	Nucleus accumbens (NAc), bilateral	Single-fraction SRS; Dmax = 30 Gy, 5-mm collimator	Voluntary alcohol consumption; MRI tissue changes (every 3 months)	1 year post-SRS	In another area, alcohol intake reduced significantly and persisted up to 9 months. The durability past 9 months was not recorded.	Animal studies have detected one case of asymptomatic increase in the size of the lateral ventricle without neurological consequences in the rest of animals.	These results constitute pre-clinical level N/A evidence due to lack of human subjects. The translation into human practice requires controlled studies in humans.
Bipolar Depression (Refractory)	Solvason et al., 2024. [86]	3	Subgenual cingulate cortex (SGC), bilateral	75 Gy, single fraction	HDRS-17 score change; clinical symptom severity	1 year; long-term follow-up in 2 patients	In the other research area, 2 out of 3 subjects improved with the mean decrease in HDRS-17 score by 27%, while 1 out of 3 did not change. The durability could not be evaluated (sample size = 3, one-time assessment).	Edema was detected in subgenual cyrus (SGC) in 2 of 3 subjects at 12 months and long term follow up. No other adverse events were recorded.	This set includes Level V evidence (n = 3 feasibility study); it may serve as a proof of concept only.

Abbreviations: GK = Gamma Knife; HDRS-17 = Hamilton Depression Rating Scale-17; NAc = nucleus accumbens; OCD = obsessive-compulsive disorder; SGC = subgenual cingulate cortex; SRS = stereotactic radiosurgery; Y-BOCS = Yale–Brown Obsessive Compulsive Scale. Level of Evidence per Oxford Centre for Evidence-Based Medicine (2009).

## Data Availability

No new data were created or analyzed in this study. Data sharing is not applicable to this article.
